# What Should Dental Services for People with Disabilities Be Like? Results of an Irish Delphi Panel Survey

**DOI:** 10.1371/journal.pone.0113393

**Published:** 2014-11-24

**Authors:** Caoimhin Mac Giolla Phadraig, June Nunn, Alison Dougall, Eunan O'Neill, Jacinta McLoughlin, Suzanne Guerin

**Affiliations:** 1 Department of Public and Child Dental Health, Dublin Dental University Hospital and Trinity College Dublin, Dublin, Ireland; 2 Public Health, Oxfordshire County Council, Oxford, Oxfordshire, United Kingdom; 3 School of Psychology, University College Dublin, Dublin, Ireland; University of Washington, United States of America

## Abstract

**Background:**

This study aimed to generate prioritised goals for oral health services for people with disabilities as a first step in meeting the need for evidence based oral health services for people with disabilities in Ireland.

**Methods:**

The study used a three round modified e-Delphi method, involving dental service professionals and people with disabilities or their representatives, in Ireland. Three rounds were completed online using SurveyMonkey. Round 1 asked: “List what you think dental services for people with disabilities in Ireland should be like.” Items for subsequent rounds were generated from responses to Round 1. Round 2 and Round 3 used 5 point Likert scales to rank these items by priority: from No Priority (1) to Top Priority (5). Consensus was achieved on each item where at least 80% of respondents considered an item either High or Top Priority. A consensus meeting concluded the process.

**Results:**

Sixty-one panelists started and 48 completed the survey. The Delphi panel agreed on level of priority for 69 items and generated 16 consensus statements. These statements covered a range of topics such as access to care, availability of information and training, quality of care, dental treatment and cost. A recurrent theme relating to the appropriateness of care to individual need arose across topics suggesting a need to match service delivery according to the individual's needs, wants and expectations rather than the disability type/diagnosis based service which predominates today.

**Conclusions:**

This process produced a list of prioritised goals for dental services for people with disabilities. This creates a foundation for building evidence-based service models for people with disabilities in Ireland.

## Introduction

About fifteen per cent of the Irish population (600,000 people in a country of just over four and a half million) report having a disability [1]. This number is expected to rise [2].

For many years research in Ireland has described poorer outcomes from oral diseases and their treatment among people with disabilities [3]–[7]. Examples of poor outcomes include extraction rather than filling of decayed teeth, increased severity and extent of periodontal disease and a lack of functional replacement of extracted teeth when people lose some teeth or become edentulous. Some possible contributing factors are evidenced in the literature, both published and gray. These include a lack of general dental practitioners who are appropriately trained and confident to meet the needs and expectations of this growing group at primary care level [8]; limited access to general anaesthesia [9], which leads to longer waiting times and poorer outcomes [10],[11]; a reported lack of resources for dental services [3] and a lack of appropriately designed service models [6]. A review of primary care dental services found inconsistent targeting of “Special Needs” groups across Ireland with great variability nationally. This report suggested a need for evidence-based service models for people with disabilities across Ireland [12].

Given the restrictions placed on healthcare systems, priorities must be selected as a first step to achieving these evidence-based service models. There is no simple, universally accepted approach to priority setting in healthcare, although it is advised that this process should be inclusive and transparent [13]. In this article we report the use of a Delphi Panel composed of representatives of stakeholders to agree on priorities for dental services for people with disabilities in Ireland.

The term Delphi Method refers to the Oracle of Delphi. It is described as a structured group communication process that allows the production of information for decision-making [14]. This allows an expert group to resolve complex problems with the goal of producing useful guidance and opinions for decision makers [15]. The Delphi method has been used in oral healthcare research for some time and is a popular and acceptable means of answering questions of clinical, educational and policy issues [15]. According to Hsu and Sanford the Delphi technique is often designed for the purpose of goal setting, policy investigation, or predicting the occurrence of future events, attempting to address “what could/should be” [16]. While Jones et al. developed oral health related goals for services through a Delphi process in a residential care setting [17] and Prabhu and colleagues [18] used a similar process to develop a clinical decision making tool for adults with disabilities, the Delphi method has not been used to identify broader goals of oral health services for people with disabilities.

### Aim

This study aimed to develop a prioritised set of goals for oral health services for people with disabilities in Ireland.

## Methods

### Design

This study used a three round modified e-Delphi. Following the three online rounds, a consensus conference was held to finalise the process.

### Ethics Statement

All participants received written information regarding their participation and provided informed consent for this study. Ethical approval was received from the Faculty of Health Sciences Research Ethics Committee of Trinity College Dublin.

### Data

File S1 contains an Excel file of summary statistics used for this analysis.

#### Sampling and participation

The sampling frame was generated using convenience sampling and snowballing. Participants were primarily invited based on their recognised standing in the fields of Public Dental Services and Disability Advocacy in Ireland. Others joined via online open registration. Participants could also recommend colleagues, thus allowing an element of snowballing. This non-anonymised registration process provided a sampling frame from which a stratified, purposive sample was selected with similar proportions of “dental” and “disability” experts to ensure a suitable range of experience and expertise within the panel. Identifiers were removed prior to analysis in successive rounds. Participants were thus “quasi-anonymised”.

#### Inclusion and exclusion criteria

The researchers felt that a broad inclusive panel would be of value and as such both dental and non-dental experts were included in the research process. The term expert refers to those who plan and provide dental services for people with disabilities as well as those who receive those dental services (and their advocates). To be considered an expert, prospective panel members had to fulfill one or more of the inclusion criteria listed in Table 1.

**Table 1:** Inclusion and exclusion criteria.

Inclusion Criteria	
1	Dental service users who have a sensory, mental, intellectual, neurological, medical, social or combined impairment that affects their oral health or access to oral health services.
2	Dental and non-dental service providers who support individuals fulfilling criterion 1 above
3	Advocates of individuals fulfilling criterion 1
4	People with experience in the delivery of dental services for people with disabilities
Exclusion Criteria	
1	Inability to participate in Delphi Process despite reasonable accommodation

### Data collection and analysis

An online secure data collection portal was used to collect responses in all three online rounds (SurveyMonkey). All responses from Round 1 to Round 3 were anonymised. A consensus conference concluded the data collection process. Details on each stage follow.

#### Round 1

We asked all panelists: “*List what you think dental services for people with disabilities in Ireland should be like.*” Items for subsequent rounds were generated from responses to Round 1. Three researchers (CM, AD, SG) then reduced these items using pre-defined principles: firstly by combining duplicate responses, secondly by coalescing responses that had the same meaning, but were expressed differently and lastly by combining low frequency items into broader topics, which encompassed multiple related items, while ensuring that their meaning was not lost.

#### Round 2

In Round 2 panelists used 5 point Likert scales to rank emergent items by priority: “Rank (the following) statements according to the level of priority that you think each should hold for dental services for people with disabilities, using the following 5 point scale”. The options included 1.No Priority; 2.Low Priority; 3.Not Sure; 4.High Priority and 5.Top Priority. The ranked mean was used to rank items by relative priority. Consensus was accepted when more than 80% of respondents gave either Top Priority or High Priority responses for an item. In order to permit continuing consideration of all items emerging from Round 1, items which failed to reach this consensus level were noted by the researchers but not removed from the materials given to panel participants until after the final consensus meeting.

#### Round 3

Statistics for all items including the median rank and level of consensus from Round 2 were fed back in a controlled manner at Round 3. Where little consensus existed on items at this stage, qualitative responses were also fed back to allow participants to consider both group statistics and qualitative responses. Panelists then rescored each item using the same Likert scale. If they felt that their score was outside of the group consensus they were encouraged to include a reason for their continuing disagreement in their Round 3 response.

#### Subgroup analysis

A subgroup analysis was undertaken for results to each item from Round 3 based on gender, urban/rural location and expert role (dental/disability). Association between category and distribution of scores on each item was tested using Pearson's chi-square statistic. Given the number of tests for each variable (n = 83), level of significance was reduced (*p*<0.025), as per the rough false discovery rate [19].

#### Consensus Conference

All participants were invited to a consensus conference. Two researchers (CM and AD) independently sorted all items arising from the e-Delphi into topics, prior to the consensus conference [20]. Next, a single statement was generated for each topic, where the meaning of constituent items were in agreement. For example, the following Consensus Statement was generated from the grouping of two items (A–B).

Consensus Statement: Oral health services should raise awareness of oral health among people with disabilities, their families, carers and non-dental health professionals (see items A and B below)

Constituent Delphi items: A. People with disabilities and their carers should be aware of the importance of oral health

B. All relevant professionals should be aware of the importance of oral health for people with disabilities

Where constituent items within a topic were contradictory or not concordant *Questions* were generated instead to encourage discussion, which ultimately led to consensus and reframing as statements. For example, the following question was generated from the grouping of two items (C–F):

Consensus Question: How often should people (with disabilities) access dental care? (see items C–F below)

Constituent Delphi items: C. Services should be structured to enable appropriate follow up

D. Services should be structured to enable regular screening and review

E. Services should be structured to enable yearly review

F. Services should be structured to enable twice yearly review

Statements and questions such as these were discussed at the consensus conference, leading to the production of 16 *Consensus Statements* as an output of this process. These were ranked by priority according to mean priority ranking of constituent items from Round 3 of the Delphi process. The number of constituent items and their mean level of agreement were also reported.

## Results

### Participant flow and sample

**Figure 1:**
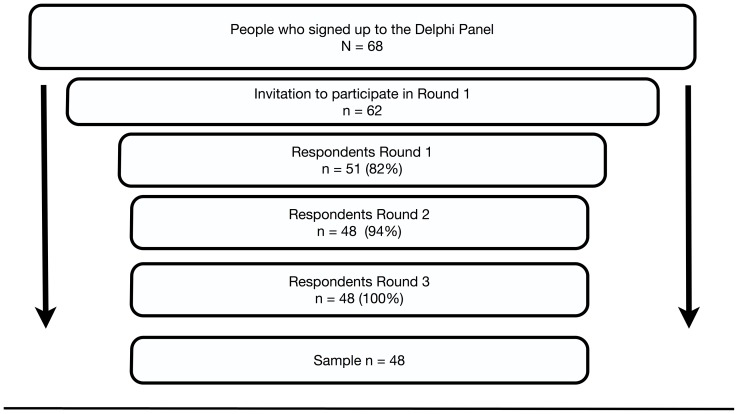
Participant Flow. Legend: N = sample completing round; % = % participants having completed the prior round, to complete the following round.

Figure 1 demonstrates the flow of participants in this study. Six people included in the sampling frame were purposively excluded: Three of these were felt not to have sufficient experience regarding dental services for people with disabilities and therefore did not meet inclusion criteria, and three others were dental professionals who were removed to reduce an over-representation of people in the category of *General Dentist (mainly public)* within the panel. Eleven panelists did not return a completed Round 1 questionnaire and were therefore eliminated from further rounds. A further three panelists, invited onto Round 2 did not respond to this survey and were also removed from further analysis.

This resulted in 48 panelists (36 females and 12 males) who completed the survey: twenty-five participants (52.1%) were dental professionals and twenty-three (47.9%) represented people with disabilities or their advocates (Table 2). Those representing dental services consisted of frontline service providers such as dental nurses, dental hygienists, private and public dentists, who often see a high number of people with disabilities, Public Dental Service managers/policy makers and specialist dental service providers such as dental public heath, paediatric and special care dentists.

**Table 2:** Professional/personal profile of panelists.

	Frequency	Percent
Total	48	100
Total representing dental services	25	52
Dental hygienist	2	4
Dental nurse	4	8
General dentist (mainly private)	4	8
General dentist (mainly public)	9	19
Manager/policy maker	2	4
Specialist dentists^1^	4	8
Total representing people with disabilities	23	48
Disability professional	14	29
Person/parent	9	19

^1^Specialists included specialist in paediatric dentistry, dental public health and special care dentistry.

The group representing people with disabilities included five people with disabilities and four parents of people with disabilities. Of the fourteen disability professionals remaining, seven were disability service providers, such as care workers and allied health professionals, four were advocates for people with disabilities (three professional and one voluntary), and two were policy makers and one a disability academic.

**Figure 2:**
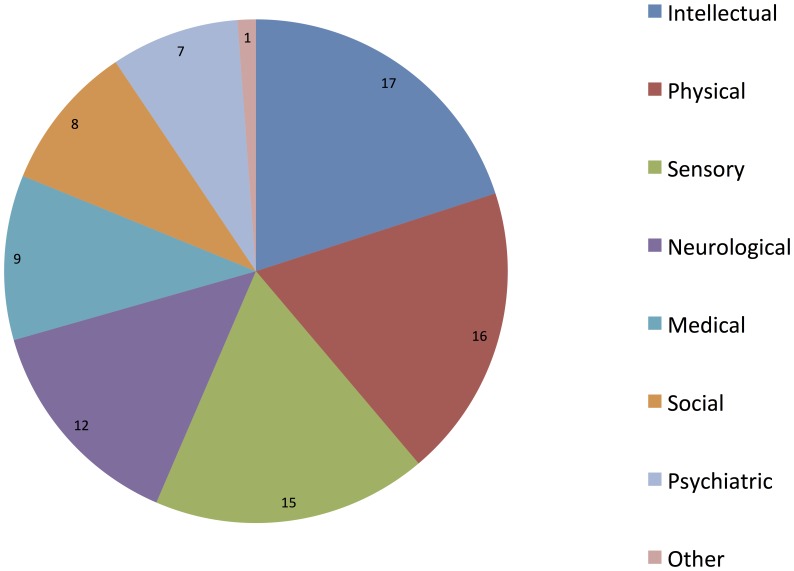
Type of disability with which panelists are associated. 27 (23 disability respondents and 4 dental) respondents reported a median of two disability types (IQR = 5) with which they were associated. Multiple categories allowed.

The panel represented a broad range of ages. Dental services panelists tended to be younger (median = 30–39 years) than those representing people with disabilities (median = 50–59 years; *p*>0.01), but the groups were otherwise similar. Geographically, participants from all four provinces of Ireland were included, although most were from the eastern seaboard (Leinster: n = 33, 68.8%). Most participants were from urban settings (n = 37, 77.1%). The panel was associated with a broad range of disability types (Figure 2).

### Data Collection and analysis

#### Round 1

A total of 351 open-ended responses were returned from 51 respondents. Following the process described above, 83 distinct items were sent back to the group for ranking in Round 2.

#### Round 2

Ninety four percent of experts completing Round 1 completed Round 2 (Figure 1). Consensus was achieved on many items at this stage: only 22 items had agreement at less than 80%. No items were considered Low or No Priority by the group, after Round 2. Forty-eight items had a median score of 5 (Top Priority), 33 a median score of 4 (High Priority) and two had a median score of 3 (Unsure). Initial rankings are presented in Tables 3 and 4.

**Table 3:** 10 items with highest mean rank at end of Round 3.

Final Rank	Item	Initial Rank in Round 2	Round 2 level of consensus (% High or Top Priority)	Round 3 level of Consensus (% High or Top Priority)	Final Median Rating in Round 3
1	Oral health services should be oriented towards prevention	8	100	100	Top Priority
1	Oral health services should be physically accessible	8	100	100	Top Priority
1	Oral health services should deliver person centred care	6	100	100	Top Priority
2	People with disabilities and their carers should be aware of the importance of oral health	3	100	100	Top Priority
3	Oral health services should be available	14	100	100	Top Priority
3	Oral health care should be responsive to individual need	11	100	100	Top Priority
3	All relevant professionals should be aware of the importance of oral health for people with disabilities	7	100	100	Top Priority
4	Oral health services should be safe for patients	1	100	98	Top Priority
4	Disability training should be provided for dental students	6	96	100	Top Priority
5	Oral health services should be accessible	14	98	100	Top Priority
5	Emergency access should be available for people in pain	4	98	100	Top Priority
5	Oral health services should enable people with disabilities, for example by maintaining the ability to eat and be comfortable	2	100	100	Top Priority
5	Oral health care should be individualised to cater for the individual needs of the person	11	100	100	Top Priority

**Table 4:** 10 items with lowest mean rank at end of Round 3.

Final Rank	Item	Rank Round 2	Round 2 level of consensus	Round 3 level of Consensus	Median Rating in Round 3	Reason For Disagreement
43	Oral health services should be totally free for all people with disabilities	43	46	28	Unsure	People with disabilities should be treated equally
42	Oral health services should be incentivised for private practitioners to encourage the treatment of people with disabilities	38	61	57	High Priority	As it is discriminatory to not see people with disabilities, therefore dentists should not be incentivised to meet their legal and professional obligations
41	Oral health services should be dependent on clearly defined scope of service	41	48	49	Unsure	Fear that this would limit services for this group
40	Oral health services should deliver care that is responsive to the diagnosis of the individual	39	57	64	High Priority	Too focused on Medical Model of disability
39	Oral health services should be free, only for those who cannot pay	44	50	72	High Priority	This would be operationally difficult
39	Oral health services should be structured to enable yearly review	38	59	72	High Priority	Risk based intervals recommended
38	Oral health services should be structured to enable Domiciliary care (home visits)	40	59	68	High Priority	May not be safe or preferable to provide specialist care in the home
38	Oral health services should be structured to enable twice yearly review	33	70	70	High Priority	Risk based intervals recommended
37	Care Pathways should be developed that are lead by local dentists in primary care settings	37	70	79	High Priority	Primary care services should receive training to act as a point of access with support from secondary services.
37	Oral healthcare for people with disabilities should only be provided in hospital settings when necessary	42	57	77	High Priority	A need for hospital based dentistry raises barriers for patients

#### Round 3

All those who completed Round 2 also completed Round 3 (Figure 1). The presence of tied ranking meant that the 83 items were ranked in 43 places. Thirty one items had 100% agreement, while 11 failed to achieve consensus at the 80% level. No items were considered Low or No Priority. Forty-eight items had a median score of 5 (Top Priority), 33 a median score of 4 (High Priority) and two had a median score of 3 (Unsure). The ten highest and lowest ranked items following Round 3 are presented in Tables 3 and 4. These tables present the ranking of specific items at Round 2 and 3 as well as the level of agreement at each stage. This gives a measure of consistency over rounds for the group as a whole. Reasons for disagreement between panelists are summarized in Table 4.

#### Subgroup analysis

Expert role and urban/rural location did not have a statistically significant association with scores on any item. The proportion of *top priority* scores was higher (*p*<0.025) among females on a number of items (*8.5); (7.1); (13.3) and (11.5)* (see Table S1 which gives further detail on all retained items and how they relate to the consensus statements.). Given the low number of participants contingency tables for the first three items listed above had more than two expected cell counts lower than 5 which limits our interpretation of these tests.

#### Consensus conference

All 83 items generated by the Delphi panel were grouped by topic as in the process described above, generating 18 statements/questions. These were reviewed by 19 panelists (7 disability and 12 dental experts) at a face-to-face conference (including 6 members of the panel who contributed remotely using online conferencing software). Following this meeting, the total number of items reduced from 83 to 69, as 11 items that had failed to reach level of consensus (80%) were now removed and 3 other items were coalesced by the consensus group. This process also reduced the number of statements/questions from 18 to 16 Consensus Statements as two statements were coalesced and one statement failed to reach the 80% cut off agreed for consensus. Table 5 summarises the final Consensus Statements generated by this round, along with their final ranking, based on mean ranking of constituent items. Included are the number of items included within each statement and their mean level of agreement.

**Table 5:** Final statements generated by Delphi Process.

Final Rank	Statement	n constituent items	Mean rank constituent items	Mean level agreement
1	Oral health services should raise awareness of oral health among people with disabilities, their families, carers and non-dental, health professionals	2	2.5	100
2	Oral health services should enable optimal outcomes for people with disabilities that meet individual need	4	8.75	99.5
3	Oral health services should be structured to enable the targeting of specific groups and deliver care based on individual need	7	10.2	98.5
4	Oral health services should be available and accessible	2	11.2	96
5	Oral health services should be designed using defined care pathways.	4	11.2	99.5
6	Oral health services should be acceptable to people with disabilities	6	12.5	98.3
7	Disability related training should be available to Dental Healthcare Professionals and students, appropriate to their need	6	12.5	96.5
8	Oral health training should be available for people, their families, carers and health professionals	6	13	99.2
9	Oral health services should be quality assured	4	15.5	97
10	Oral health services should be structured to enable frequency of care, appropriate to individual need	4	16.5	95.5
11	A range of Oral health services including emergency, preventive, primary and secondary care, should be available as appropriate to individual need	8	17.7	96.5
12	Oral healthcare should be available within an acceptable timeframe	2	19.5	95.5
13	Oral health services should be well resourced	4	21.7	93.5
14	Information and documentation should be accessible, and available in suitable formats where appropriate	4	26.2	91
15	Care pathways should be developed that allow people to choose Oral healthcare settings, appropriate to individual need	8	26.8	88.3
16	Oral health services for people with disabilities should be integrated both with general Oral health and non-Oral health services	7	27.4	93.7

Statements relate specifically to oral health services for people with disabilities. This is implied in most statements to reduce burden except where this phrase is needed for clarity. The statement: *Services should be accessible locally* (initial ranking 8, n = 3, mean rank of included items = 17, mean level of agreement = 94%) was removed during this consensus meeting and amalgamated with the Statement 2 as contributors felt that this represented needless repetition; The statement *Novel funding models of oral health service for people with disabilities should be examined* (initial ranking 17, n = 3, mean rank of included items = 41, mean level of agreement = 52%) was removed at the end of the consensus conference as all constituent items failed to achieve the agreed level of consensus of 80%.

## Discussion

Structured engagement with service users is important when setting priorities for health services [13]. This article reports a novel means of identifying priorities using structured engagement – in this case with a single broadly constituted sample. These priorities were generated in response to an identified need for evidence-based service models [12]. The study met its aim by generating a list of 16 consensus statements regarding oral health services for people with disabilities, as well as a complementary ranked list of 69 priority items.

The topics, which arose in this study, were diverse encompassing access to care, quality of care, dental treatment, information and cost. Many items were ranked as top or high priority by the Delphi Panel. This must be kept in mind when considering the ranking outcome. There was very little difference in mean rank between items or statements. Although their final ranking may suggest an ordinal relationship, often the difference between ranked items was minimal. Meaningful ranking is therefore difficult, as previously reported [20]. Nevertheless, it was perhaps surprising that the highest priority statement to arise from this process related to health awareness. This highlights the importance of “thinking outside of the box” when considering goals for dental services, especially where resources may limit potential for achieving alternative priorities.

Many items mirrored a multidimensional model of access to care as described by Penchansky [21], though an added dimension relating to *appropiateness of care to individual need* arose. Previous research found this additional dimension specific to dental services for people with disabilities [22],[23]. Many statements focused on meeting individual need rather than focusing on diagnosis. This element of care delivery recognised the importance of focusing on the individual, their expectations, desires and needs when accessing and receiving care and prevention, rather than a disability or disease specific focus. This is in line with the World Health Orgnaisation's *International Classification of Functioning, Disability and Health* (ICF) model of disability [24] which is increasingly applied to understand, measure and improve awareness of oral health for people with disabilities [19],[25],[26]. This focus was evident across Delphi panel statements suggesting that individual need should drive the individual's frequency of contact with oral health services, the choice of healthcare setting, the availability of care pathways, and types of service available and accessible (such as general or specialist, community or hospital). Individual need should also decide optimal outcomes from interactions with oral health services for the individual.

### Methodological issues

Traditional Delphi approaches emphasise the importance of group interaction on the development of consensus, yet this study relied mainly on interaction online. An approach to address the potential lack of group interaction is to incorporate formal feedback within the e-Delphi Process [27]. The authors were also conscious to avoid undue coercion for participants to cede to group pressure. Therefore responses were anonymous to other participants with a focus on statistical group responses. Controlled feedback was also provided at each stage [28]. In Round 2 participants received the list of items grouped by topic for their ranking exercise and in Round 3, feedback was provided to participants as the median level of priority and level of consensus for each item following Round 2. This allowed participants to consider the group scoring before final ranking without undue pressure to conform [28]. Participants could also provide a reason for disagreeing with the group consensus at this stage if they wished.

Despite the broad representation on this panel, we should not misinterpret the panel as being “representative” in the true sense. Sampling was non-probabilistic and therefore we were not surprised that the group does not reflect the natural occurrence of particpants seen in society. For example this panel contained twice as many public dentists than private practitioners while in reality approximately 80% of Irish Dentists are based in private practice. This has implications for how we intepret the results.

The concept of disability used in this process was broad. This was considered favourable to accommodate a broad range of views across perpectives rather than the views of a specific disability subgroup. An outcome of this inclusive approach is that the general concepts which were prioritised by this group may be different to the specifc priorities of groups of people with specific disabilities (e.g. people with communication disabilities compared to people with physical disabilities or bleeding disorders). Therefore the results of this Delphi panel could be used as a starting point for further validation with specific groups, where indicated.

Despite reasonable accommodation, the nature of this study meant that some people with communication and or cognitive difficulties could not be included. Additional processes were initiated for those with communication or cognitive issues and these are reported elsewhere. People with visual impairment were included using Braille documents with appropriate formatting of questionnaires and associated documentation.

The retention rate in this suvey was good at each stage. Most losses occurred in the initial round where 17.7% of those selected did not respond. Only a further 5.8% did not complete the following two rounds. This is a pleasing result as retention over rounds is a recognised problem in Delphi Panel research. Importantly, this means that no participant dropped out of later rounds due to disagreement with the group consensus. It is felt that the use of email, and online data collection was a large part of this success. Additionally, the short timeframe between rounds was also felt to benefit the response rate. One challenge the team encountered was the need to generate Braille versions of questionnaires. Given the quick turnaround, this addition challenged both researchers and respondents, but doubtlessly enriched the data collected.

Delphi panel results are often improved by including diverse perspectives on a problem [29]. Therefore this study included experts from a broad range of backgrounds, most importantly, people with disabilities and their advocates. This is in acknowledgement of their “expertise by virtue of having experienced the impact of a condition or intervention” ([29], p. 379). A larger panel is obviously needed to incorporate sufficient numbers of people with these diverse opinions, which are known to increase the number of items generated and also reliability [30]. In the absence of clearly accepted guidance on panel sizes [31], this study aimed to include 60 panelists, giving equal representation of disability and dental experts. Forty eight panellists completed all rounds, giving equal representation of disability and dental experts. Interestingly, the subgroup analysis did not find any statistically significant difference between dentists and disability experts. Only gender showed significant difference on a small number of items, the reason for which is unclear.

The consensus conference led to the development of common topics among clustered items, a useful technique, to make it conceptually easier for participants (who were at this stage also knowledge users) to consider large sets of items concisely. This clustering by topic has previously been used for similar purposes [20]. The consensus conference enabled the group to clarify and agree meaning within statements and items. The research team felt that this step was beneficial. While it was well attended, a self-selecting subgroup of the Delphi Panel attended this step and this may have influenced the resultant output. Online attendance was evidently favoured by some participants who could not attend, thus allowing broader attendance.

#### Implications

This study reveals a clear focus on placing the indvidual at the centre of services. The clear, recurrent emphasis on delivering services according to *individual needs and expectations* rather than based on diagnosis is poignant. This means that services should be planned and delivered to meet the needs of the individual with disabilities in an acceptable manner. This may present challenges to those who plan and deliver services. Further research is needed to conceptually understand individual need among this group, and operationally, how this is presently and can be met.

We have used the outcomes of this Delphi to produce research tools for application with people with specific disability types to help understand their health care priorities and adapt the results of the current study. These results will be reported seperately. The research team will use the results of these initial studies to develop local priorities for people with disabilities and oral health services in Ireland. This will hopefully lead to theory development upon which to design service models.

Previous research has questioned whether priorities produced by public opinion surveys are relevant or useful for decision-makers [32] and the results of this Delphi survey may similarly be questioned, especially if one attempts to infer from an Irish context to other areas. While poor oral health among people with disabilities appears to be a global phenomenon [33],[34], it is unlikely that the priorities for dental services will be the same internationally. Similar processes could be undertaken in other countries within the policy, practice and cultural norms of other societies.

Lastly, it is hoped that policy makers and commissioners can also learn from our experiences when planning services for this group of patients. The intention is that policy makers can consider the consensus statement of interest with the option of mining the constituent items that may give guidance as to the goals of the services which they may commission. A measure of agreement and priority will enrich the utility of these data. A full list of items is available in Table S1.

## Conclusions

This study places a clear focus on the indvidual at the centre of oral health services. Services should be planned and delivered to meet the needs of the indivdual with disabilities in a way that is accpetable to that person. The study represents a first step in the development of future policy, service development and research in this area of public health and special care dentistry. As the items and statements summarised in this article represent a set of priorities as agreed by one panel of experts, there is a need to incorporate other opinions to truly achieve consensus on the question: What should dental services for people with disabilities be like? As such, the research team has undertaken qualitative work with specific groups of people with disabilities to develop broader consensus using the outcomes described here as a foundation. The research team aim to ultimately use this process to develop interventions for evaluation in Ireland. The pursuit of these goals will surely engage those who wish to rise to this challenge.

## Supporting Information

Table S1 **Project SMILE Ireland Delphi Panel consensus statements and constituent items.** Consensus statements are presented in order of priority along with mean rank and level of agreement. Constituent items are similarly presented with rank and level of agreement and agreed priority. Statements relate specifically to oral health services for people with disabilities. This is implied in most statements to reduce burden except where this phrase is needed for clarity. The statement: Services should be accessible locally (initial ranking 8, n = 3, mean rank of included items = 17, mean level of agreement = 94%) was removed during this consensus meeting and amalgamated with the Statement 2 as contributors felt that this represented needless repetition; The statement Novel funding models of oral health service for people with disabilities should be examined (initial ranking 17, n = 3, mean rank of included items = 41, mean level of agreement = 52%) was removed at the end of the consensus conference as all constituent items failed to achieve the agreed level of consensus of 80%.
(XLSX)

File S1 **Excel file of summary statistics used for this analysis.**
(XLSX)
